# Preliminary Phytochemical Screening and *In Vitro* Anti-*Helicobacter pylori* Activity of Extracts of the Stem Bark of *Bridelia micrantha* (Hochst., Baill., Euphorbiaceae)

**DOI:** 10.3390/molecules16086193

**Published:** 2011-07-25

**Authors:** Benjamin I. Okeleye, Pascal O. Bessong, Roland N. Ndip

**Affiliations:** 1 Microbial Pathogenicity and Molecular Epidemiology Research Group, Department of Biochemistry and Microbiology, Faculty of Science and Agriculture, University of Fort Hare, P/Bag X1314, Alice 5700, South Africa; E-Mail: 200803960@ufh.ac.za (B.I.O.); 2 AIDS Virus Research Laboratory, Department of Microbiology, University of Venda, Thohoyandou 0950, South Africa; E-Mail: pascal.bessong@univen.ac.za (P.O.B.); 3 Department of Biochemistry and Microbiology, Faculty of Science, University of Buea, Box 63, Buea, Cameroon

**Keywords:** *Helicobacter pylori*, *Bridelia micrantha*, minimum inhibitory concentration, rate of kill, phytochemicals

## Abstract

*Helicobacter pylori* is a major risk factor for gastritis, ulcers and gastric cancer. This study was aimed to determine the antimicrobial activity of the stem bark of *Bridelia. micrantha* on *H. pylori* isolated in South Africa. Extracts and clarithromycin were tested against 31 clinical strains, including a standard strain (NCTC 11638) of *H. pylori*, by measuring the diameters of the corresponding inhibition zones, followed by determination of the Minimum Inhibitory Concentration (MIC) (using metronidazole, and amoxicillin as control antibiotics) and the rate of kill. Preliminary phytochemical screening was also done. Inhibition zone diameters which ranged from 0–23 mm were observed for all five of the extracts and 0–35 mm for clarithromycin. Marked susceptibility of strains (100%) was noted for the acetone extract (P < 0.05), followed by ethyl acetate extract (93.5%). The MIC_50_ values ranged from 0.0048 to 0.156 mg/mL for the ethyl acetate extract and 0.0048 to 0.313 mg/mL for the acetone extract. The MIC_90_ values ranged from 0.0048 to 2.5 mg/mL for the ethyl acetate extract and 0.078 to >0.625 mg/mL for the acetone extract, respectively. Insignificant statistical difference in potency was observed when comparing the crude ethyl acetate extract to metronidazole and amoxicillin (P > 0.05). Complete killing of strain PE430C by the ethyl acetate extract was observed at 0.1 mg/mL (2 × MIC) and 0.2 mg/mL (4 × MIC) at 66 and 72 h. For strain PE369C, 100% killing was observed at 0.1 mg/mL (2 × MIC) in 66 and 72 h. The ethyl acetate extract could thus be a potential source of lead molecules for the design of new anti-*Helicobacter pylori* therapies as this study further confirmed the presence of phytochemicals including alkaloids, flavonoids, steroids, tannins and saponins.

## 1. Introduction

*Helicobacter pylori* is one of the most common chronic bacterial pathogens of humans. It colonizes the gastric epithelial surface and withstands the stomach’s hostile environment by microaerophilic growth capacity and the production of numerous virulence factors which may lead to chronic gastritis, peptic ulceration and gastric cancer in later life [1,2]. Infections in the developing countries have been reported to be higher than in developed countries. In Africa, 70 to 80% are infected with the organism and 61 to 100% harbour the organism in sub-Saharan Africa [3,4,5].

Treatment could be achieved by a combination of therapeutic agents such as antibiotics, bismuth subsalicylate, proton pump inhibitors and H2-blockers. However, treatment failure rates remain as high as 5 to 20%, along with frequent relapses of gastric ulcers and the cure achieved is incomplete and undesirable side effects are known to occur [6]. The organism has developed resistance against most antibiotics, especially metronidazole, which therefore limits their use in the treatment of infections and this problem is being encountered more in Africa [4,7]. Other factors including poor patient compliance, the significant cost of combination therapy, and the non-availability of medications in rural areas, especially in Africa, reveals the need to develop alternative approaches to suppress/cure the infection.

Plant materials have been reported to be present in or have provided the models for about 50% of Western drugs, with herbal remedies demonstrating encouraging results in the cure of many diseases [8]. Medicinal plants such as *Thymus kotschyanus, Ageratum conyzoides* (Linn), *Scleria striatinux* (De Wild) and *Lycopodium cernua* (Linn) Pic. Serm have shown potential anti-*Helicobacter pylori* activities [8,9].

*Bridelia micrantha* Hochst., Baill. (Euphorbiaceae), also known as Coast gold leaf in English (Munzere in Venda) is a semi-deciduous to deciduous tree, up to 20 m tall, with a dense rounded crown and tall, bare stem, widespread in South Africa. The stem bark has been traditionally employed in South Africa to treat several clinical conditions, including intestinal parasites, gastritis, salmonellosis and gastro-enteritis, stomach problems, human immunodeficiency virus/acquired immune deficiency syndrome (HIV/AIDS), infertility, neurosis and psychosis [10,11]. This plant has been reported to possess compounds like friedelin, alpha-amyrin, gallic acid, luteoforol and also being active against *E. coli*, *K. pneumoniae*, *S. flexneri*, *P. aeruginosa* and other organisms [12].

To the best of our knowledge, this plant has not been evaluated for its antimicrobial activity on clinical isolates of *H. pylori* isolated in South Africa; an organism with profound heterogeneity compounded by an emerging trend of resistance to the current treatment regimen employed in South Africa [7]; hence the search of potential sources of easily available starting materials for the synthesis of new drugs against the pathogen.

## 2. Results and Discussion

### 2.1. Anti-Helicobacter pylori Activities

It was observed in our results that the zones of inhibition ranged from 12 to 20 mm for the ethyl acetate extract; 16 to 23 mm for the acetone extract; 0 to 15 mm for the water extract and 0 to 35 mm for clarithromycin (Table 1 and Table 2).

**Figure 1:**
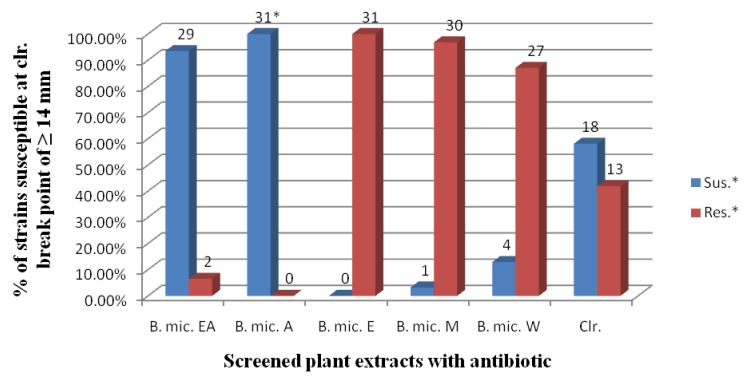
Plant extracts and antibiotic susceptibility against 31 strains of *H. pylori*.

One hundred percent susceptibility was noted for the acetone extract. Susceptibility to the ethyl acetate, methanol and water extracts were 93.5%, 3.2%, and 12.9%, respectively. For the control antibiotic, clarithromycin it was 58.1% (Figure 1). The mean difference of the acetone extract of *B. micrantha* was statistically significant (P < 0.05) compared to the other extracts and clarithromycin at 95% Confidence Interval (Table 2).

For the MIC determination, an inhibition zone of ≥14 mm was chosen as representative of bacterial susceptibility to the extracts (acetone and ethyl acetate) and antibiotic. Ethyl acetate (20%) and DMSO (10%) used as negative control, showed no activity.

The high prevalence of *H. pylori* infection as well as the increasing trend of antibiotic resistant strains to the current treatment regimen in the Eastern Cape Province of South Africa has been reported, but few studies have examined the activities of medicinal plants against the pathogen in Africa in general and South Africa in particular [7,8,13]. We are not aware of any study which evaluated the anti*-H. pylori* activity of *B. micrantha* in spite of its profound antimicrobial potential against several microorganisms, including *Campylobacter* spp., a close relative to *H. pylori* [10,12,14].

The present study revealed that the ethyl acetate extract demonstrated inhibition zones against this pathogen that ranged from 12 mm to 20 mm and for acetone, 16 mm to 23 mm (Table 2). This is in line with similar zone diameters of inhibition for ethyl acetate and other solvent extracts of some medicinal plants from Cameroon against *H. pylori* [8]. However, Adeleye *et al.* [15] reported a diameter of 0 mm zone of inhibition for the water extract of the leaves of this plant. In the present study, we documented a zone diameter of 0 mm to 15 mm. This may be due to the fact that the different parts of the plant may contain different phytochemicals.

The acetone (100%) and ethyl acetate (93.5%) extracts were observed to be the most active against all the *H. pylori* strains tested, compared with the other solvents used in our study (Figure 1). This is similar to the results of other investigators [16,17], who equally reported marked activity of ethyl acetate extracts compared to other solvents. Djipa *et al*. [18] and Asha *et al.* [19] reported potent antimicrobial activity of an acetone extract against pathogenic bacteria compared to other solvents used. This may be due to the fact that the active compounds against *H. pylori* strains in the plant were less polar, semi-polar solvents dissolve semi-polar compounds best and different solvents extract different compounds [8].

### 2.2. MIC determination at MIC50 and MIC90

The MIC_50_ values for *B. mic.* EA ranged from 0.0048 to 0.156 mg/mL; 0.0048 to 0.313 mg/mL for *B. mic* A, and 0.0048–0.156 for metronidazole and amoxicillin respectively. The MIC_90_ for *B. mic.* EA ranged from 0.0048 to 2.5 mg/mL; 0.078 to >0.625 mg/mL for *B. mic* A; 0.0098 to >5 mg/mL for metronidazole and 0.078 to >2.5 mg/mL for amoxicillin (Table 3).

**Figure 2:**
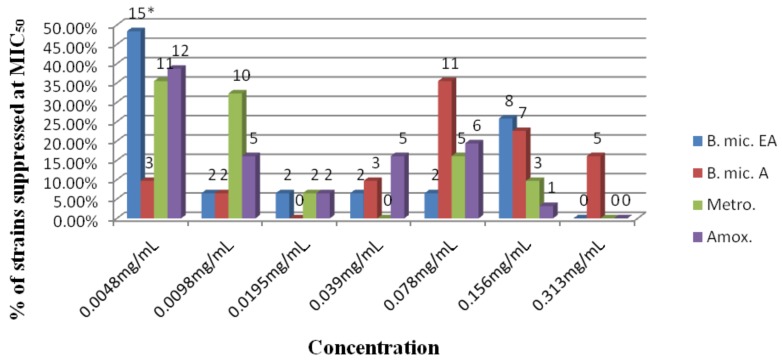
Antibacterial profile (MIC_50_) of the tested extract with two antibiotics (metronidazole and amoxicillin) against 31 strains of *H. pylori*.

One hundred percent of the strains were suppressed by *B. mic.* EA, followed by amoxicillin, 12 (38.7%) and metronidazole, 11 (35.5%) at a concentration of 0.0048 mg/mL (at MIC_50_). At 0.078 mg/mL, 11 (35.5%) strains were inhibited by *B. mic* A followed by amoxicillin, 6 (19.4%) and metronidazole, 5 (16.1%) (Figure 2). The activity of *B. mic.* EA was not statistically significant (P > 0.05) to the two antibiotics tested. However, a statistically significant difference in potency was observed for *B. mic.* A compared to the two antibiotics (P < 0.05), but not between metronidazole and amoxicillin (P > 0.05).

The average MIC_50_ values of the extract in this study, which ranged from 0.0048 to 0.156 mg/mL, was 0.052 mg/mL (Table 3). Samie *et al*. [12], found that *B. micrantha* was mostly active against *S. flexneri* (1.5 mg/mL) and most of the other organisms tested with a MIC value that ranged between 3 to >12 mg/mL. This was less active compared to our current result which may be attributed to the different organisms used, solvent used in extraction, season in which the plants were collected as well as storage conditions amongst others, as all these factors have been reported to affect the antimicrobial activity of plants [20,21].

For all the isolates tested, the MIC values of the antibiotic ranged from 0.0048 mg/mL (4.8 µg/mL) to 0.156 mg/mL for amoxicillin and metronidazole, respectively, which is similar to a recent study in the same locality conducted by Tanih *et al.* [7] who reported 2.5 µg/mL–5.0 µg/mL for amoxicillin. Metronidazole and amoxicillin which served as the positive control however, had no statistically significant difference (P > 0.05) observed at 95% Confidence Interval in activity compared to *B. micrantha* ethyl acetate extract.

### 2.3. Rate of Kill

**Figure 3:**
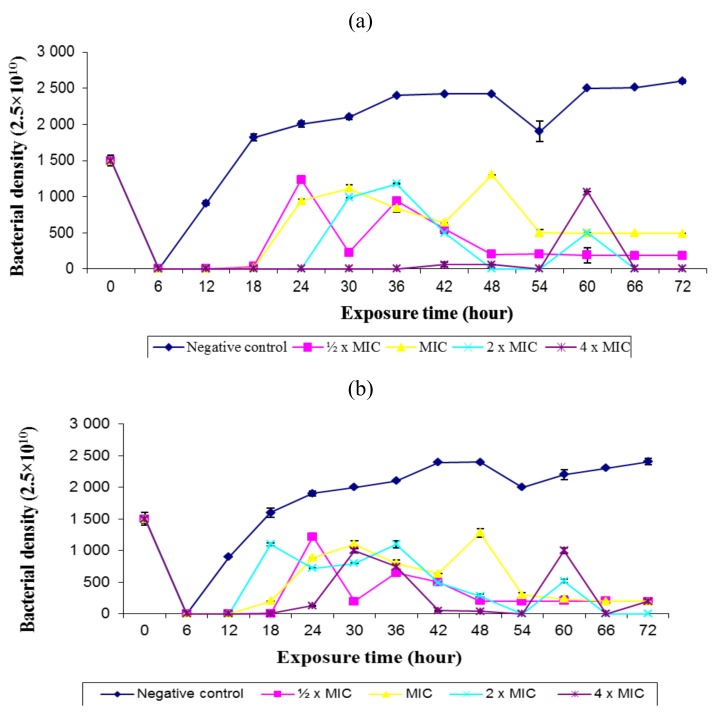
Profile of rate of kill of *H. pylori* strain (**a**) PE430C and (**b**) PE369C by ethyl acetate extract of *Bridelia micrantha* stem bark compared to untreated strain (negative control).

Time course of the extract at different concentrations was examined. *B. mic*. EA completely inhibited the growth of *H. pylori* strain PE430C at 0.025 mg/mL, 0.05 mg/mL, 0.1 mg/mL and 0.2 mg/mL in 12 h and at 0.1 mg/mL and 0.2 mg/mL in 18 and 24 h of incubation, respectively. Growth and inhibition were later observed from 30 to 60 h before 100% killing at 0.1 mg/mL and 0.2 mg/mL after 66 and 72 h. Like strain PE430C, strain PE369C was totally inhibited at all the concentrations in 12 h. Inhibition was also observed at 0.1 mg/mL and 0.2 mg/mL in 54 and 66 h. One hundred percent killing was however observed at 0.1 mg/mL (2 × MIC) in 66 and 72 h (Figures 3a and 3b).

Apart from five strains with MIC90 > 0.5 mg/mL, others ranged from 0.0048 to >0.156 (Table 3). This is in line with the highest killing effect observed at a concentration of 0.2 mg/mL (4 × MIC) (Figures 3a and 3b), which corroborates the study of Ali *et al.* [22]; and more potent compared to the observation reported in other studies [23,24]. The organism was completely killed when the exposure time was increased to 66 and 72 h. From the results, the rate of kill exhibited by the extract against the test strains appeared to be both concentration and time dependent, which is in agreement with previous observations [22,24].

### 2.4. Phytochemical Compounds

Qualitative phytochemical analysis revealed the presence of flavonoids, steroids, tannins, alkaloids, and saponins. It was observed from the reactions (colours, heamolysis, turbidity, layers, emulsification and precipitation) that there may be much more of the steroids, tannins, flavonoids, and saponins present in the ethyl acetate extract of *B. mic*. EA compared to alkaloids (Table 4).

This plant part (stem bark) was observed in this study to possess the common compounds present in many active plants, which have been reported by many investigators to be high in antimicrobial properties [25]. These biologically active phytochemical compounds contribute to the antimicrobial activities of many plants. For example, the importance of tannins for the treatment of inflamed or ulcerated tissues has been reported together with remarkable activity in cancer prevention and anticancer conditions symptomatic of *H. pylori* infection [24]. Flavonoids in the human diet reduce the risk of various cancers [26], while saponins are known to produce an inhibitory effect on inflammation [11]. Alkaloids have been reported to have analgesic, anti-spasmodic and bactericidal effects [25]. Ali *et al.* [22] had also documented compounds from *Eugenia caryophillis* and *Cinnamomum verum*, including eugenol and cinnamaldehyde, respectively, which at a concentration of 2 μg/mL completely inhibited all their *H. pylori* strains. It may therefore be the effects of these compounds that are responsible for the activities observed in the ethyl acetate extract of *B. micrantha* tested in the present study.

## 3. Experimental

### 3.1. Bacterial Strains

Thirty-one strains of *H. pylori*, in addition to a control strain NCTC 11638, were subjected to antimicrobial assays in this study. Strains were isolated from patients presenting with gastric related morbidities at the Livingston Hospital, Port Elizabeth for endoscopy and confirmed following our previously reported scheme [4]. Informed consent was obtained from the patients and ethical approval (Protocol number EcDoH-Res 0002) from the Eastern Cape Department of Health, and the institutional review board of the University of Fort Hare (GMRDC).

### 3.2. Preparation of Plant Extracts

*Bridelia micrantha* (stem bark) was selected based on ethnobotanical information. The plant was collected and identified in Limpopo Province, South Africa in collaboration with a botanist at the University of Venda where voucher specimens (number BP03) have been deposited. The extraction method described by Ndip *et al*. [8] was employed. Briefly, the plant was harvested and processed. Technical grade ethyl acetate, acetone, ethanol, methanol (100%) and water were employed for extraction. The samples were dried in an oven (30–40 °C) and milled before extraction. Dried plant material (2 kg) was macerated in the solvent and the slurry was put in a shaker incubator (Edison, N.J. U.S.A.) regulated at room temperature (RT) for 48 h then centrifuged (Model TJ-6 Beckman, U.S.A.) at 3,000 rpm for 5 min. The combined extracts (exhaustive extraction) were concentrated in a rotavapor (Buchi R461, Switzerland) and a 6.2 g sample of each plant extract was used for the preliminary bioassay. Stock solutions were prepared in 20% ethyl acetate for *B. mic.* EA extract and 10% dimethyl sulphoxide (DMSO) for other extracts, which we established to be non inhibitory to *H. pylori*.

### 3.3. Screening of Crude Extracts for Anti-H. pylori Activity

The agar-well diffusion method was used in accordance with the method previously described with some modifications [27]. *H. pylori* inoculum was prepared from subcultures of bacteria in sterile distilled water and the turbidity adjusted to 1.5 × 10^8^ CFU/mL (corresponding to 0.5 McFarland standards). It was evenly inoculated on Brain Heart Infusion (BHI) agar (Oxoid, U.K.) supplemented with 7% horse blood (Oxoid) and Skirrow’s supplement (Oxoid) and allowed to dry for 3 to 5 min. Wells were punched in the plates using a sterile stainless 6 mm cork borer. The wells were filled with 30 µL of the extract (50 mg/mL). DMSO (10%) and ethyl acetate (20%) were used as negative controls and 0.05 µg/mL clarithromycin as a positive control. The tests were repeated in duplicate and incubated microaerophilically at 37 °C for 72 h (Anaerocult Basingstoke, U.K.). *H. pylori* control strain NCTC 11638 inoculated plate was included in all the experiments.

### 3.4. Determination of Minimum Inhibitory Concentration (MIC_50_ and MIC_90_)

The MIC was carried out in accordance with the method of Banfi *et al*. [28] with modifications. Extracts that gave a zone of inhibition ≥14 mm were chosen for MIC determination by the microdilution test method in 96-well plates. Two-fold dilutions of the most potent extracts (ethyl acetate and acetone) and antibiotics, metronidazole and amoxicillin were prepared in the test wells in complete BHI broth supplemented with 7% horse serum and Skirrow’s supplement; the final extracts and antibiotics concentrations ranged from 0.0048–10 mg/mL respectively and the tests were carried out in duplicate. Each strain applied into the wells was serially diluted to correspond to 0.5 McFarland standards and incubated under microaerophilic condition at 37 °C for 3 days. After incubation, resazurin solution was added per well and further incubated at 37 °C for 1 h. It was read with a microtiter plate reader adjusted to 620 nm (Model 680 Bio-Rad, Japan) and the MIC_50_ and MIC_90_ were determined.

### 3.5. Determination of the Rate of Kill

The time kill assay of bacterial isolates by *B. mic.* EA (which gave the best potent activity) was carried out as described by Ali *et al*. [22] with little modification. The test organism was standardized to 10^8^ CFU/mL and an aliquot from each strain suspension (0.5 mL) was added to BHI broth (4.5 mL) supplemented with 7% horse serum and Skirrow’s supplement and the solution of the extracts (½ × MIC, MIC, 2 × MIC and 4 × MIC) added. These were incubated at 37 °C in a microaerophilic cabinet shaking at ~120 rpm over a period of 72 h at 6 h interval (0, 6, 12, 18, 24, 30, 36, 42, 48, 54, 60, 66, 72 h). The experiment was performed in duplicate. An exact volume (0.5 mL) of each suspension was withdrawn at time intervals and transferred to BHI broth recovery medium (4.5 mL) containing 3% “Tween 80”. The suspension was then serially diluted and plated out for viable counts. The control plates contained the bacterial cells without the extract.

### 3.6. Phytochemical Analysis

Adopting the methods of Adegboye *et al*. [25], the qualitative phytochemical analysis of the ethyl acetate extract of *B. micrantha* was carried out to test for alkaloids, flavonoids, tannins, steroids and saponins.

### 3.7. Statistical Analysis

Analysis was performed using the SPSS Version 17.0 (Illinois USA, 2009). The one way ANOVA test was used to determine if there was any statistically significant difference in the diameter of zones of inhibition of the plant extracts and clarithromycin; the MIC of the most active extracts (ethyl acetate and acetone) and the control antibiotics (metronidazole and amoxicillin). P-values <0.05 were considered significant.

## 4. Conclusions

This study demonstrated the *in vitro* inhibitory and bactericidal activity of the crude extracts of *B. micrantha* bark. The plant may provide novel or lead compounds, which could become template for the synthesis of new anti-*H. pylori* drugs. Isolation and characterization of the bioactive compounds would be our major focus in future studies.

## Figures and Tables

*, No. of strains; * Sus, Susceptible; * Res, Resistance.

* No. of strains.

**Table 1:** Antibacterial activity of plant extracts against *Helicobacter pylori* strains.

Zone diameter of inhibition of growth (mm)						
*H. pylori strains*	*B.mic.* (EA)	*B.mic.* (A)	*B.mic.* (E)	*B.mic.* (M)	*B.mic.* (W)	Clr.
PE11A	16	22	0	0	8	10
PE26A	17	22	8	8	10	18
PE93A	18	20	0	0	0	28
PE93C	18	20	0	8	9	8
PE102C	16	20	0	0	11	16
PE115A	17	20	0	0	11	10
PE155A	16	20	0	0	9	15
PE162A	12	22	0	8	10	35
PE219C	18	20	0	0	11	12
PE252C	20	20	0	0	11	15
PE258C	15	21	8	8	10	10
PE265C	16	22	0	0	9	13
PE296C	14	21	0	0	9	0
PE308C	19	21	0	0	10	15
PE369A	13	20	0	12	13	20
PE369C	16	20	7	11	12	18
PE402A	14	22	0	11	15	12
PE406C	16	21	7	10	13	27
PE407C	17	23	0	10	12	13
PE411C	15	20	0	0	13	17
PE430A	18	18	10	0	12	21
PE430C	17	19	0	8	15	31
PE436A	14	16	0	0	10	23
PE436C	15	22	8	8	14	20
PE462A	15	21	8	12	13	18
PE462C	16	21	8	8	10	17
PE466C	17	17	0	10	13	25
PE467A	16	20	7	10	14	0
PE467C	16	20	0	12	13	0
PE469C	17	20	7	8	13	8
PE471A	16	21	7	14	9	0

*B.mic.*: *Bridelia micrantha*; EA: Ethyl acetate; A: Acetone; E: Ethanol; M: Methanol; W: Water; Clr.: Clarithromycin. The data is the average of duplicate observations.

**Table 2:** Mean zones and inhibition diameter range of the crude extract and antibiotic.

Extract/control antibiotic	Mean zone diameter (mm)	Inhibition diameter range
*B.mic.* (EA)	16.13 ± 1.708	12–20 mm
*B.mic.* (A)	20.39 ± 1.476	16–23 mm
*B.mic.* (E)	2.74 ± 3.794	0–10 mm
*B.mic.* (M)	5.68 ± 5.108	0–14 mm
*B.mic.* (W)	11.03 ± 2.811	0–15 mm
Clr.	15.32 ± 8.852	0–35 mm

*B.mic.*: *Bridelia micrantha*; EA: Ethyl acetate; A: Acetone; E: Ethanol; M: Methanol; W: Water; Clr.: Clarithromycin. Data are mean ± SD values of 31 determinations for each extract and antibiotic.

**Table 3:** *In-vitro* anti-*H. pylori* activities of *B. micrantha* ethyl acetate and acetone extracts and antibiotics at MIC_50_ and MIC_90_ (MIC mg/mL).

	*B.mic.* (EA)		*B.mic.* (A)		Metronidazole		Amoxicillin	
*H. pylori* strains	MIC_50_	MIC_90_	MIC_50_	MIC_90_	MIC_50_	MIC_90_	MIC_50_	MIC_90_
PE11A	0.156	>0.625 *	0.078	0.313	0.156	0.625	0.156	ND *
PE26A	0.078	>0.156	0.078	0.313	0.156	>0.625	0.039	>0.313
PE93A	0.039	ND	0.078	ND	0.0098	ND	0.039	ND
PE93C	0.0048	>0.0048	0.078	>0.313	0.0195	ND	0.0048	>0.625
PE102C	0.156	>0.625	0.078	0.313	0.078	0.156	0.039	0.156
PE115A	0.0195	ND	0.313	ND	0.0098	ND	0.0098	ND
PE155A	0.0048	>0.0048	0.156	ND	0.0048	ND	0.0048	0.313
PE162A	0.0048	>0.0048	0.078	>0.625	0.0048	ND	0.0048	0.078
PE219C	0.0048	>0.0048	0.0048	>0.156	0.0048	ND	0.078	0.313
PE252C	0.156	ND	0.313	ND	0.0098	ND	0.0098	ND
PE258C	0.156	2.5	0.078	0.313	0.078	0.156	0.078	0.156
PE265C	0.0048	>0.0048	0.156	ND	0.0048	ND	0.0048	1.25
PE296C	0.0048	0.0048	0.0048	0.625	0.0098	0.625	0.0048	0.156
PE308C	0.0048	>0.0048	0.078	ND	0.0048	ND	0.0048	>0.625
PE369A	0.156	ND	0.156	ND	0.078	>5	0.078	>2.5
PE369C	0.0098	>0.156	0.156	ND	0.0098	ND	0.0098	ND
PE402A	0.156	>1.25	0.078	0.625	0.156	>0.156	0.078	>0.156
PE406C	0.078	ND	0.039	ND	0.0098	>5	0.0195	ND
PE407C	0.156	1.25	0.078	0.313	0.078	>0.156	0.039	0.625
PE411C	0.0048	>0.0048	0.313	ND	0.0098	ND	0.0048	0.156
PE430A	0.0048	ND	0.313	>0.625	0.0195	ND	0.039	0.156
PE430C	0.156	>0.156	0.156	ND	0.078	>5	0.078	>0.313
PE436A	0.0048	>0.156	0.0098	>0.078	0.0048	ND	0.0048	ND
PE436C	0.0048	0.0048	0.0098	0.625	0.0048	0.0098	0.0048	0.625
PE462A	0.039	ND	0.313	ND	0.0098	ND	0.0098	ND
PE462C	0.0195	ND	0.039	ND	0.0098	ND	0.0195	ND
PE466C	0.0098	ND	0.156	ND	0.0098	ND	0.0098	ND
PE467A	0.0048	>0.0098	0.039	>0.078	0.0048	ND	0.0048	0.625
PE467C	0.0048	>0.0048	0.156	ND	0.0048	ND	0.078	0.313
PE469C	0.0048	0.0048	0.0048	0.313	0.0048	1.25	0.0048	0.313
PE471A	0.0048	>0.0048	0.078	>0.313	0.0048	ND	0.0048	0.313
Average	0.052		0.118		0.034		0.031	

* ND, Not determined; *>, Closer but not exact.

**Table 4:** Qualitative analysis of phytochemicals in the ethyl acetate extract of *Bridelia micrantha*.

Phytochemical	*Bridelia micrantha* (ethyl acetate extract)
Alkaloids	++
Flavonoids	+++
Steroids	+++
Tannins	+++
Saponins	+++

++, moderately present; +++, strongly present.
